# The Fly a Carrier of Micro-organisms

**Published:** 1891-01

**Authors:** Henry Bartens

**Affiliations:** St. Louis


### THE FLY A CARRIER OF MICRO-ORGANISMS.

BY HENRY BARTENS, A. M., M. D., ST. LOUIS.

The question whether all infectious diseases are produced by micro-organisms, or, whether the micro-organisms are the product of disease, is still open to discussion. One fact, however, is firmly established, to wit: that micro-organisms do really exist, and that they may enter the body, producing septic infection and death.

Micro-organisms may enter the living body through lesions of the skin, openings of sweat pores, hair follicles, or through lesions of the mucous membranes, openings of the ducts and follicles, pockets, sulci and folds.

At this time, I would call your attention to the fact that the common fly is frequently the medium of septic infection; and shall try to illustrate this assertion by citing some of my observations made in practice.

The first case of this nature came into my hands about four years ago, when I practiced in Concordia, Mo. An old lady, the wife of a farmer, had been infected by a fly, which had fed upon the carcass of a dead hog lying in the vicinity of her house. The fly alighted on her neck, she crushed it with a stroke of her hand, and thought no more of it until about three days later, when a most painful inflammatory swelling on the neck recalled the incident. Unfortunately, she was advised to poultice the swelling (the worst thing she could do under the circumstances), and it was only when matters became very serious that she sent for medical aid. I found an immense suppurating carbuncle situated on her neck ; herself in a half comatose condition with all the evidence of blood poisoning. I made in this case several free incisions into the swelling to afford an outlet to the accumulated matter, and dressed it with compresses soaked in a five per cent. carbolized solution, but in spite of my efforts she died—the fly had done its deadly work. Right here I would make a protest against poultices of all kinds. Never apply a poultice to a carbuncle or any inflammatory swelling caused by septic infection, but always apply cloths wrung out of a warm antiseptic solution. They will accomplish more in one hour than the old fashioned dirty poultice will do in a week.

The next case came under my observation in August, 1888, immediately after my removal to Nokomis, Ill. There, I was called to see a young butcher, suffering from a swollen arm, high fever and severe symptoms of depression. This is the history of this case : Three days previously, he had been working with bare arms among the rubbish at the slaughter-house, when all at once a whole swarm of flies alighted on his arm—flies which had been feasting on the bones and refuse generally found in such places. In driving them off his arm, he crushed a number of them, and forgot the fact until three days later, when his arm became, without any visible cause, painful and swollen, causing a most serious illness. In this case, I could not prevent suppuration, although I used the most rigid antiseptic measures and most careful internal treatment, and he recovered only after a very protracted illness.

The third and most remarkable case is that of a fisherman living on the Illinois river, in Calhoun county. It was in the fall of 1888, when, in consequence of the low water and other unknown causes, a great many fish perished. They were thrown ashore, filling the air with their stench, and thousands of flies feasted upon them. This man was working on his nets at the time, barefooted and barearmed as such such men generally do—all around him flies were buzzing, and now and then one would select his bare legs or arms as a suitable resting place.

The man occasionally drove them away, scratched his legs when he felt an itching, but otherwise paid no attention to them. A few days later, he commenced to feel very ill, a number of boils formed on his extremities, and he called in a physician, but died from blood poisoning ultimately.

How could all these persons be septically infected without there being any lesion of the skin? There is but one explanation: The micro-organisms were deposited by the flies upon the skin, and entered through the open sweat pores. The fly is our enemy, and he who will find ways and means to destroy him effectually, will be a benefactor to mankind.

